# Supplementary material for: Mapping the pathways of photo-induced ion migration in organic-inorganic hybrid halide perovskites
Source: Nat Commun. 2023 Apr 3;14:1846. doi: 10.1038/s41467-023-37486-w (PMC10070404; doi:10.1038/s41467-023-37486-w)
Supplement: Supplementary file 1 — Supplementary Information [file 41467_2023_37486_MOESM1_ESM.pdf]

Supplementary Information: Mapping the Pathways of Photo-induced Ion Migration in Organic-inorganic Hybrid Halide Perovskites

Taeyong Kim,[1,2] Soyeon Park,[3] Vasudevan Iyer,[4] Basamat Shaheen,[1]
Qi Jiang,[3] Usama Choudhry,[1] Gage Eichman,[4] Ryan Gnabasik,[1]
Kyle Kelley,[4] Benjamin Lawrie,[4,5,*] Kai Zhu,[3,†] and Bolin Liao[1,‡]

[1]*Department of Mechanical Engineering,*
University of California, Santa Barbara, CA 93106, USA
[2]*Department of Mechanical Engineering,*
Seoul National University, Seoul, 08826, Republic of Korea
[3]*National Renewable Energy Laboratory, Golden, CO 80401, USA*
[4]*Center for Nanophase Materials Sciences,*
Oak Ridge National Laboratory, Oak Ridge, TN 37830, USA
[5]*Materials Science and Technology Division,*
Oak Ridge National Laboratory, Oak Ridge, TN 37830, USA

(Dated: March 15, 2023)

* lawriebj@ornl.gov
† kai.zhu@nrel.gov
‡ bliao@ucsb.edu

SUPPLEMENTARY NOTE 1: ADDITIONAL DATA

A. XPS, XRD and PL Characterization Data

To confirm the quality of our samples, we conducted X-ray photoelectron spectroscopy (XPS) characterization of our $FAPbI_3$ and $MAPbI_3$ samples to determine the relative atomic ratio. XPS detects photoelectrons from the very surface of the sample and thus does not rely on modeling the transport process inside the sample. The measured electron binding energy corresponding to Pb $4f$ and I $3d$ levels are shown in Fig. S1. Based on the XPS spectra averaged over several locations on the samples, the iodine-to-lead ratio is estimated to be 4.1 in $FAPbI_3$ and 3.3 in $MAPbI_3$. The small deviation from the nominal ratio of 3 might be due to slight sample surface degration during sample shipping from NREL to UCSB, which is consistent with a previous study[1]. We further provide the X-ray diffraction data in Fig. S2 and the photoluminescence data in Fig. S3 that confirm the high quality of the samples.

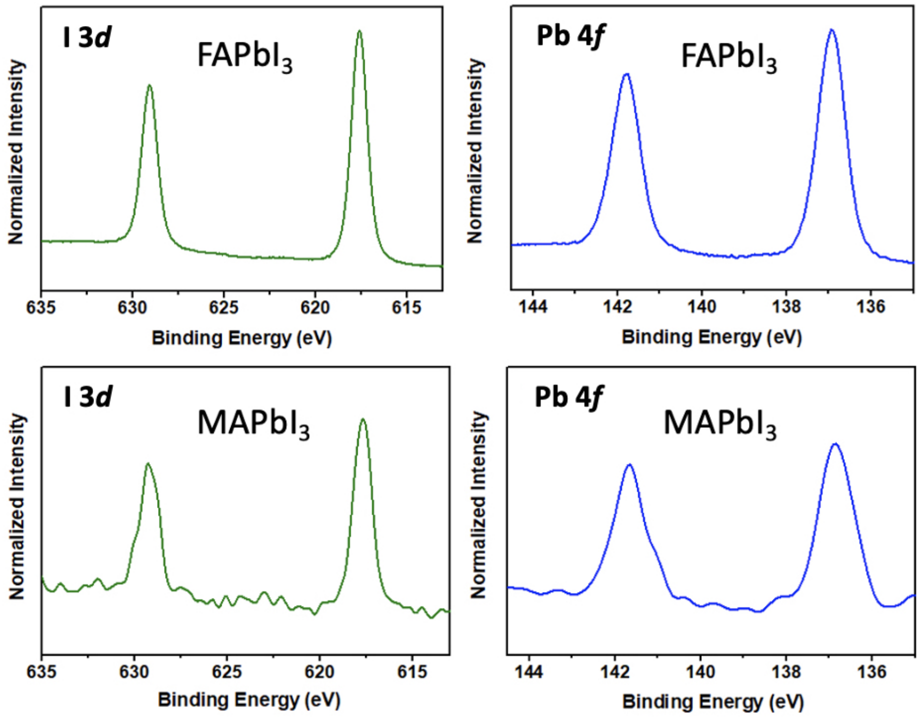

Figure S1. High-resolution XPS spectra of I $3d$ and Pb $4f$ levels in perovskite thin films. The top panels are the spectra for $FAPbI_3$ and the bottom panels are the spectra for $MAPbI_3$.

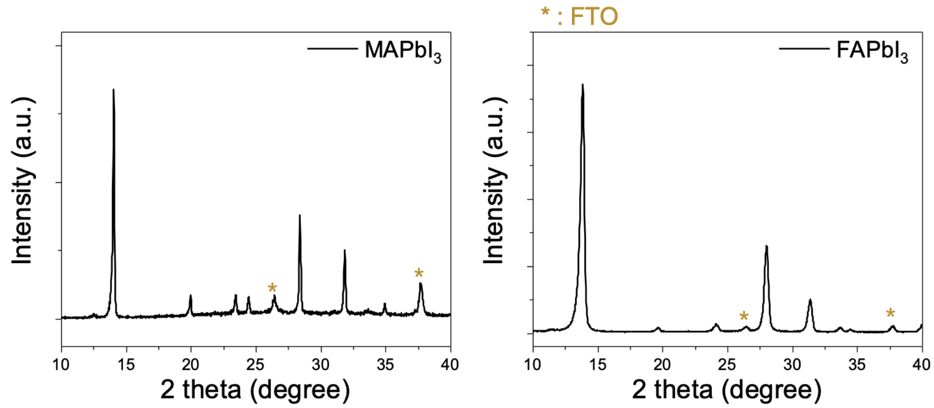

Figure S2. XRD scans of our $MAPbI_3$ and $FAPbI_3$ samples. The stars label the peaks associated with the FTO substrate.

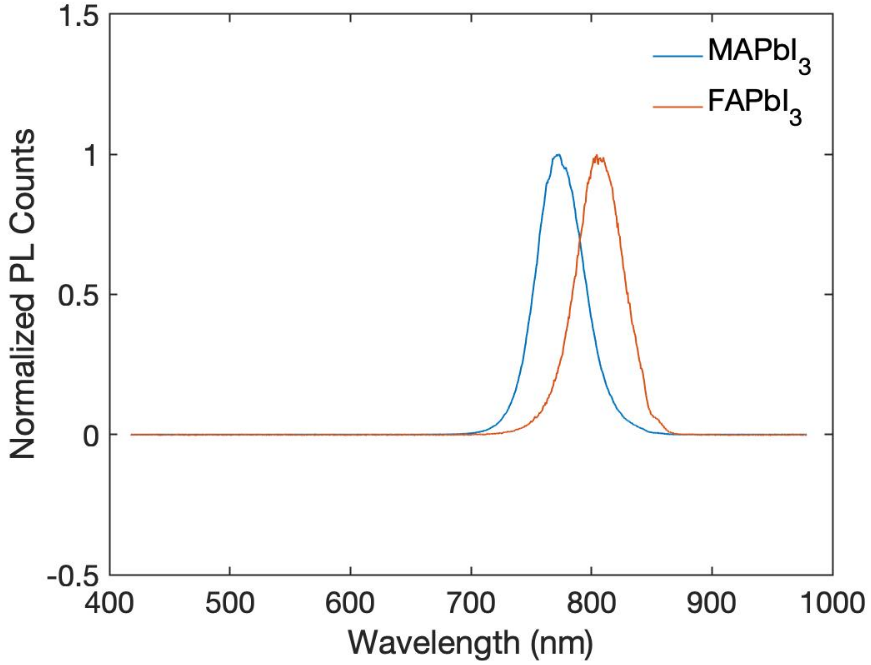

Figure S3. Photoluminescence Characterization of Our $MAPbI_3$ and $FAPbI_3$ samples. A continuous 532-nm excitation laser was used with 2 μW power and 200 ms collection time.

B. EDS spectra for pristine $MAPbI_3$

Additional EDS spectra measured at primary electron (PE) energy of 30 keV for pristine $MAPbI_3$ is shown here.

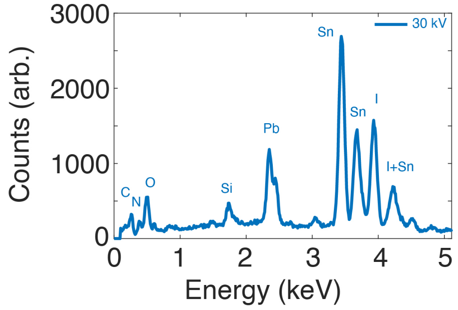

Figure S4. EDS spectrum of pristine $MAPbI_3$. Shown is collected X-ray counts versus photon energy at PE energy of 30 keV in pristine $MAPbI_3$ without light exposure. Detected elements are annotated near each characteristic peak in the figure.

C. EDS spectra at other PE energies for pristine $MAPbI_3$

In this section, additional EDS spectra measured at other PE energies are presented. The measured datasets are given in Fig. S5. The magnitude of the characteristic peaks due to the elements in the substrate (tin and silicon) decreases as the PE energy decreases, indicating that the corresponding EDS spectra depends on the penetration of the PEs through the depth of the sample.

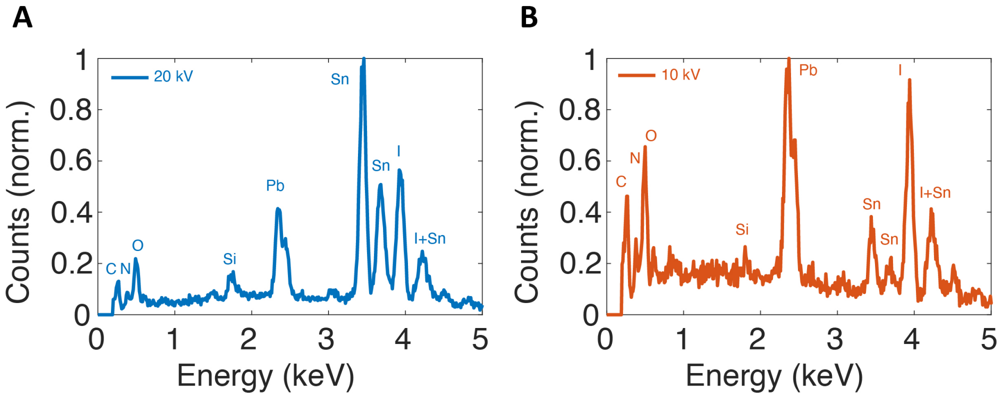

Figure S5. Additional EDS emission spectrum at other PE energies for pristine $MAPbI_3$. Measured EDS spectra at PE energies of (A) at 20 keV, and (B) at 10 keV. Detected elements are annotated near each characteristic peak in each figure.

D. Additional SE Images with Different Optical Power

Light exposure tests with lower optical power compared to those reported in the main text were conducted and the results are shown here. Bright contrasts were still observed in both $FAPbI_3$ and $MAPbI_3$ with a reduced optical power. In the case of $FAPbI_3$, no bright-ring-shaped contrast was formed with the reduced optical power up to 3-hour exposure.

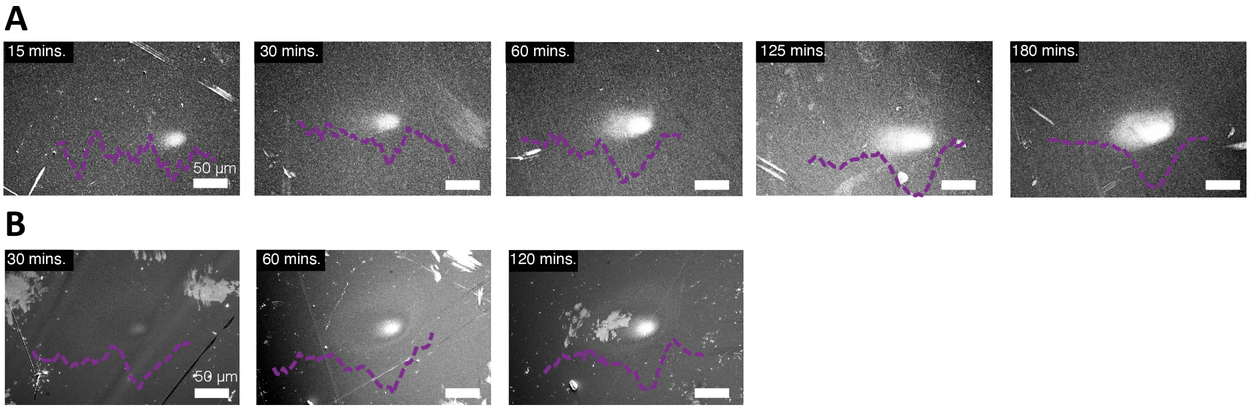

Figure S6. Additional SE images and EDS horizontal line profiles of iodine at different optical fluences and exposure times. (A) Measured data taken in $FAPbI_3$ at optical power of $\sim$ 2.5 mW (optical fluence: $\sim$ 39.8 μJ cm^{-2}), and (B) those in $MAPbI_3$ at optical power of $\sim$ 4.1 mW (optical fluence: $\sim$ 66.0 μJ cm^{-2}). Exposure time is labeled in the images.

We also took high-resolution SEM images after the light exposure of $FAPbI_3$ to examine the microstructural change after light exposure. A representative image is shown here in Fig. S7. Consistent with the observations shown in Fig. 5 in the main text, the area exposed to light has smaller grains compared to the pristine area. The bright "ring"-shaped edge area has a width of a few micrometers and is likely due to an increased electrical resistance that leads to a local accumulation of charge. An AFM morphology scan of the light exposed area is shown in Fig. S8, which also confirms the smaller grains within the light-exposed area.

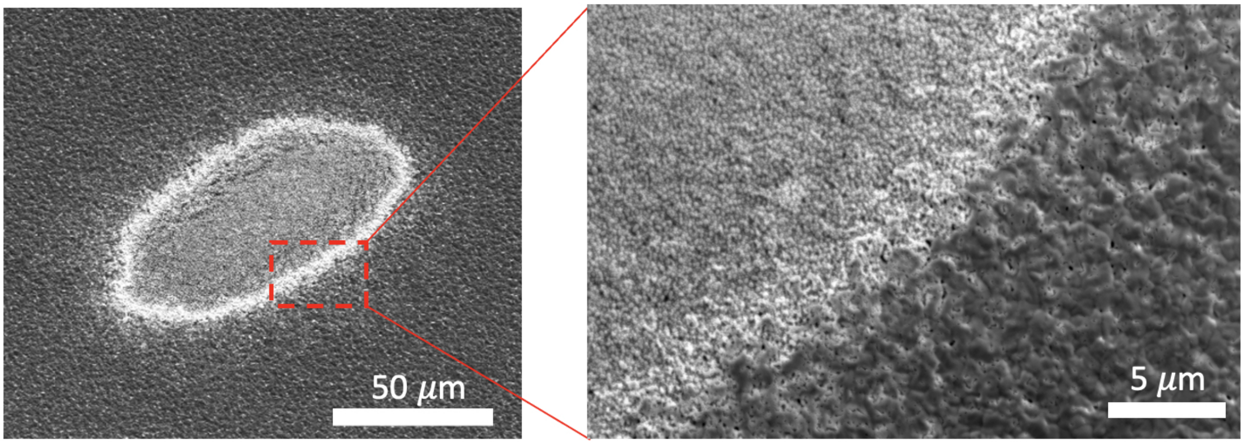

Figure S7. High-resolution SEM images of $FAPbI_3$ after light exposure, showing the microstructural change. The sample was exposed to light with a fluence of 105 μJ cm^{-2} for 30 minutes. 5-keV PEs were used to generate the images. It is clearly shown that light-exposed area has smaller grains compared to the pristine area. The bright "ring"-shaped edge of the exposure area is most likely due to an increased electrical resistance that leads to local charge accumulation.

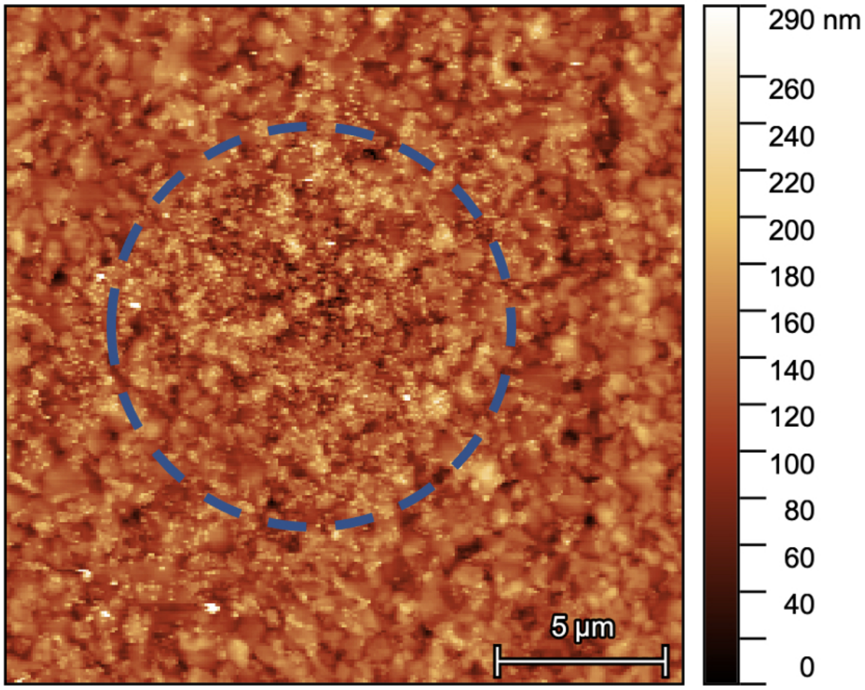

Figure S8. AFM morphology scan of $FAPbI_3$ after light exposure, showing the microstructural change. The sample was exposed to light with a fluence of 105 μJ cm^{-2} for 30 minutes. The light exposure area is denoted by the blue dashed circle. The AFM image shows smaller grains within the light-exposed area.

E. Irreversibility of the photo-induced contrast change

Due to the iodine deficiency near the surface that can be caused by I_2 escaping to the vacuum, we do not expect the observed ion migration effects to be reversible. To confirm it, we conducted additional tests by monitoring the change in the light-induced secondary electron contrast over time. The results are shown here in Fig. S9. Figure S9A shows the secondary-electron contrast in $FAPbI_3$ induced by light exposure (5 minutes, fluence: 106.6 μJ cm^{-2}). Then the laser was turned off and the sample was stored inside the SEM vacuum chamber (vacuum level: 10^{-6} torr) up to 12 hours. The secondary-electron contrast after 4 hours, 10 hours and 12 hours are shown in Fig. S9B-D, indicating the contrast remains the same within this period of time and the photo-induced change is irreversible.

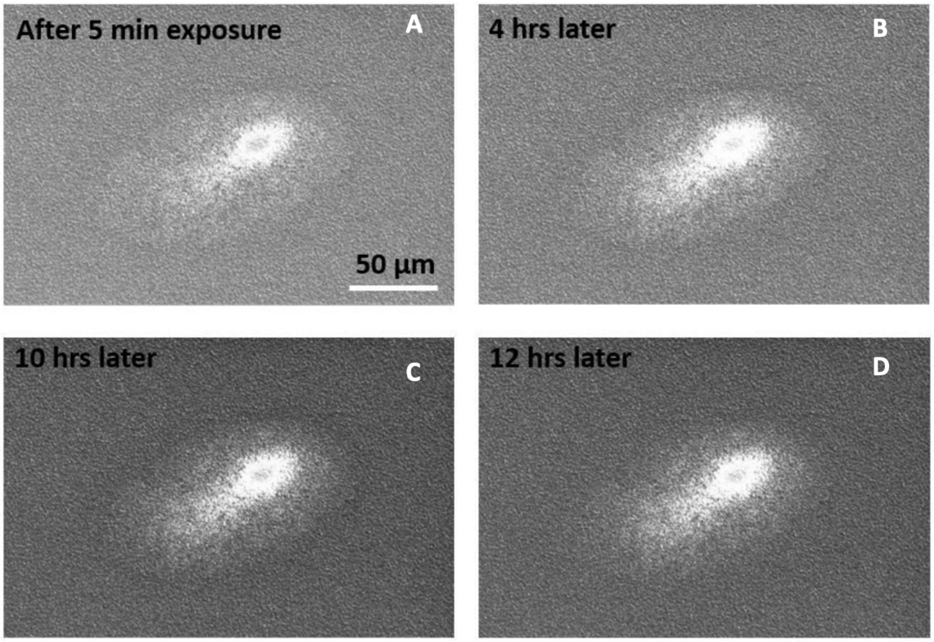

Figure S9. Reversibility test of the photo-induced secondary electron contrast in $FAPbI_3$. A: The photo-induced secondary electron contrast in $FAPbI_3$ after light exposure for 5 minutes (fluence: 106.6 μJ cm^{-2}). B-D: the secondary-electron contrast after storing the sample in the vacuum chamber for 4 hours, 10 hours and 12 hours, respectively. The contrast induced by 5-minutes light exposure remains after 12 hours, suggesting the effect is not reversible spontaneously.

F. Additional EDS linescan results for $FAPbI_3$ and $MAPbI_3$

As discussed in the main text, we performed the EDS linescan experiments in $FAPbI_3$ and $MAPbI_3$ at various optical powers, exposure times, and PE energies. The corresponding additional data is presented in Figs. S10, S11, S12. We note that an artifact near horizontal location of ~ 100 μm can be seen in Fig. S12, which is due to a morphological defect on the scanned area in $MAPbI_3$.

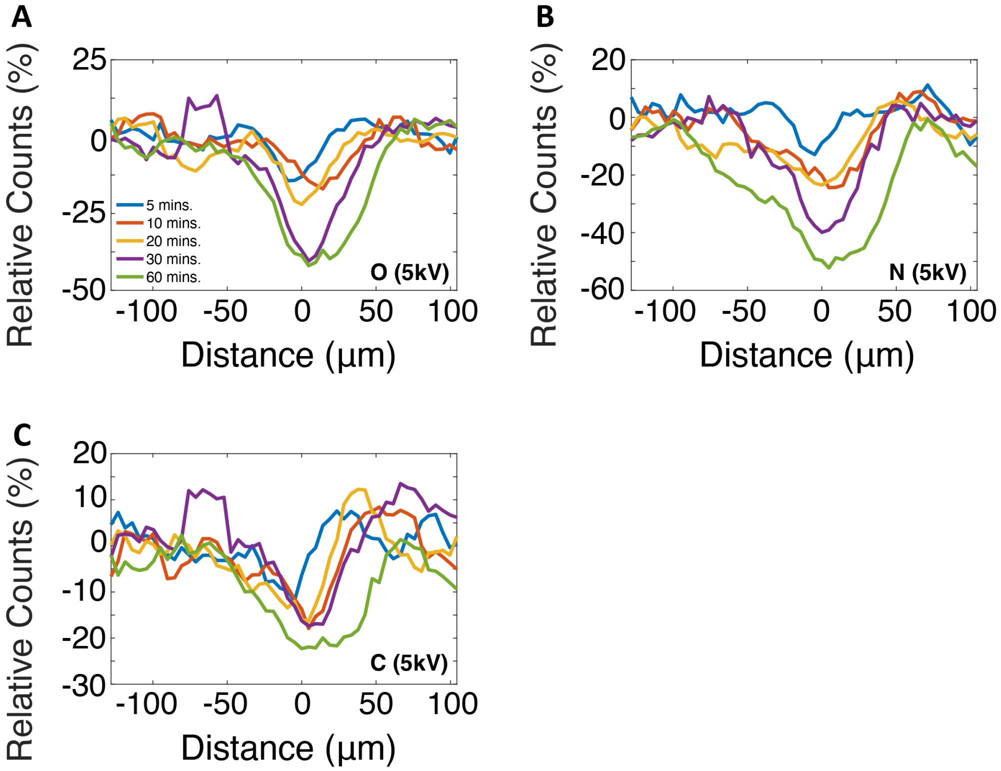

Figure S10. Additional EDS linescan results in $FAPbI_3$ at optical power of 6.7 mW, corresponds to an optical fluence of ~ 106.6 μJ cm^{-2}. Measured X-ray counts versus distance from the center of the optical beam for (A) O (5-keV PE energy), (B) N (5-keV PE energy), and (C) C (5-keV of PE energy). Legends indicate the optical exposure time. A reduction of these elements suggest a reduced concentration of the organic cations near the surface in the illuminated area, indicating photo-induced decomposition of the organic cations.

Additional EDS horizontal line profile for silicon at 5-keV PE energy under various optical exposure times and optical powers is shown in Fig. S14. At 5-keV PE energy, despite the presence of detected small trace of silicon, their distribution did not change appreciably due to the light exposure, suggesting the migration of silicon ions mainly occurred deep inside the bulk sample.

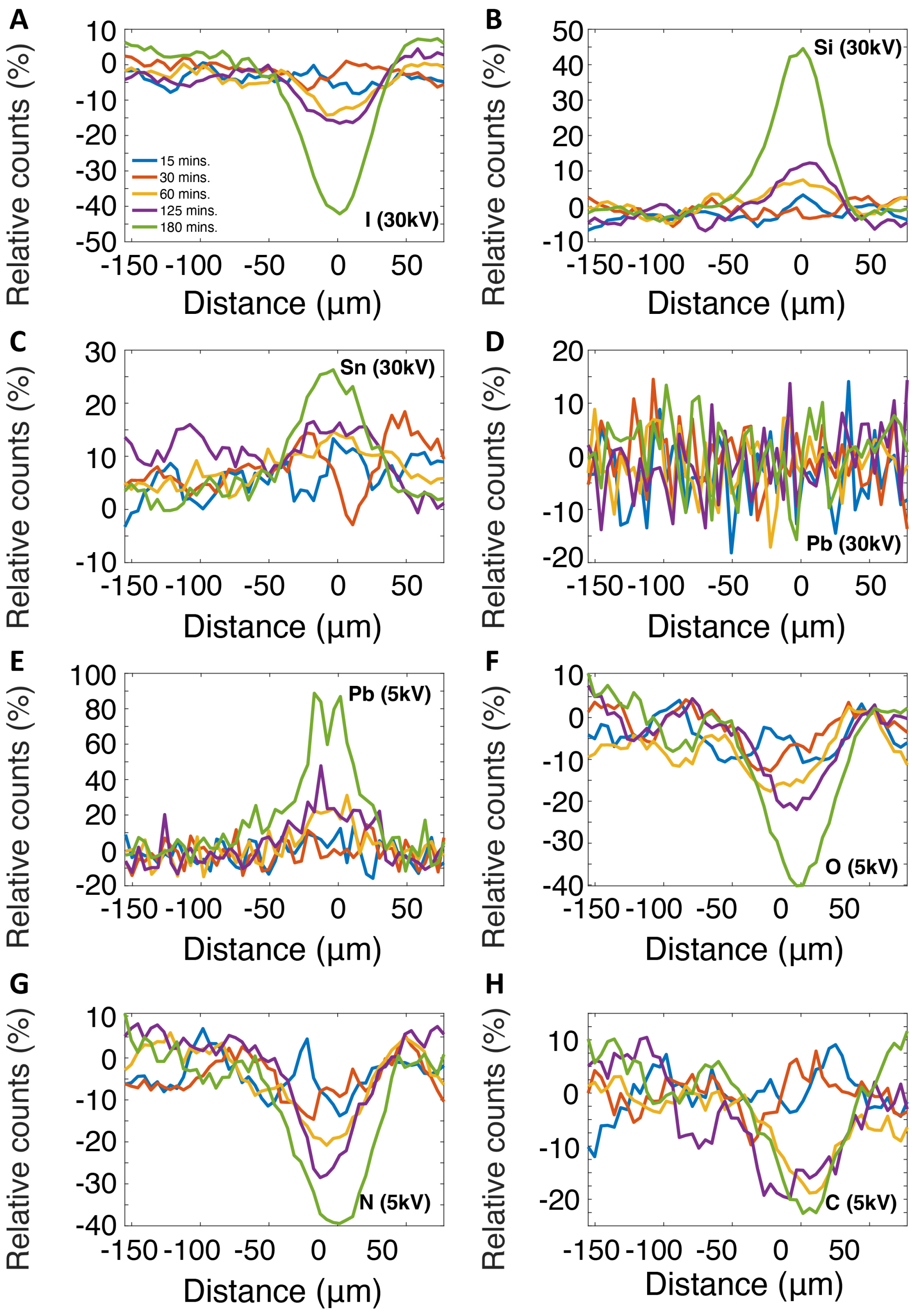

Figure S11. Additional EDS linescan results in $FAPbI_3$ at optical power of 2.5 mW, corresponding to an optical fluence of $\sim 39.8\ \mu$J cm^{-2}. Measured X-ray counts versus distance from the center of the optical beam for (A) I (30-keV PE energy), (B) Si (30-keV PE energy), and (C) Sn (30-keV PE energy), (D) Pb (30-keV PE energy), (E) Pb (5-keV PE energy), (F) O (5-keV PE energy), (G) N (5-keV PE energy), and (H) C (5-keV PE energy). Legends indicate optical exposure time. The qualitative features of the ion distributions are similar to the case with a higher optical fluence that is discussed in the main text.

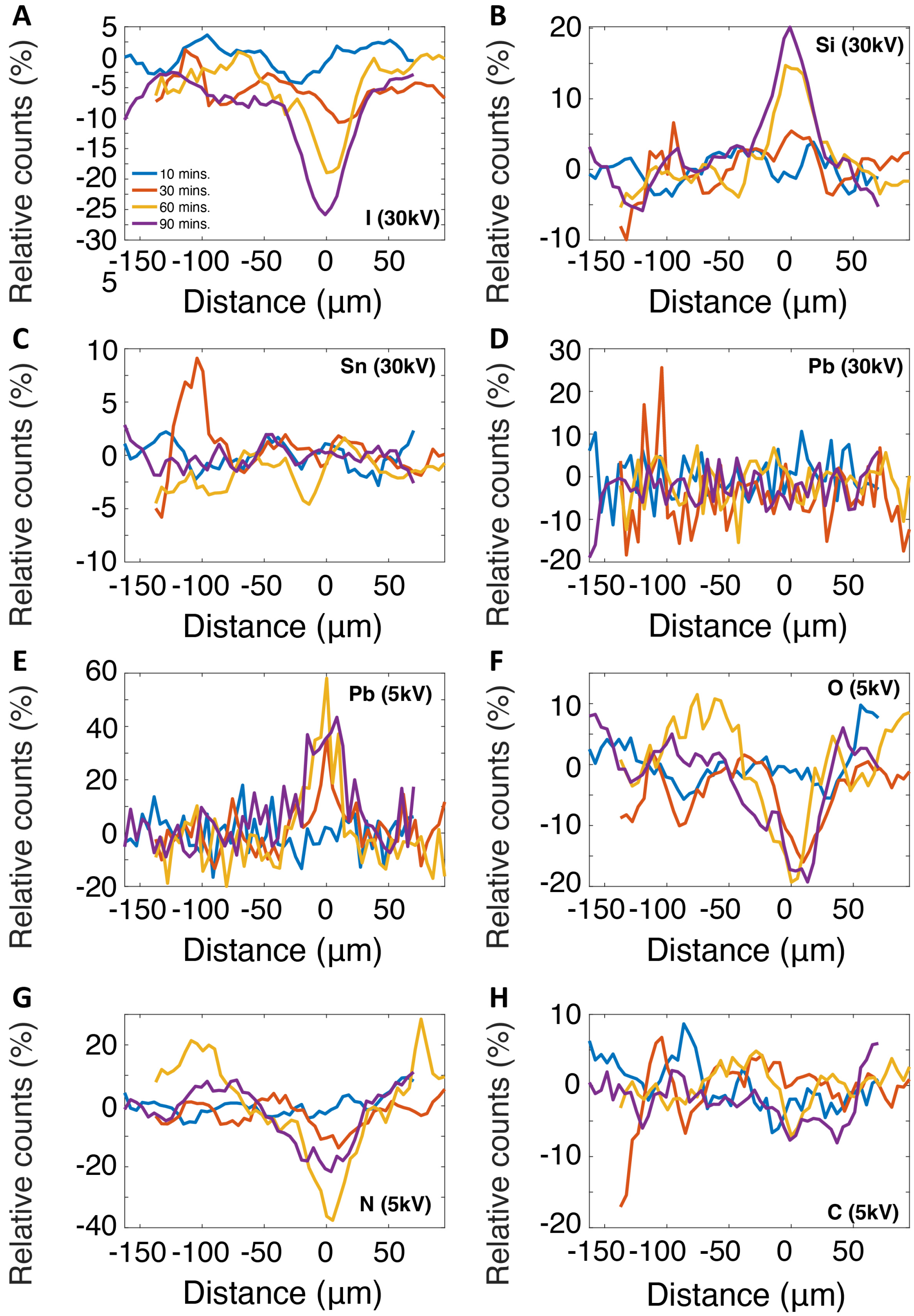

Figure S12. Additional EDS linescan results in $MAPbI_3$ at optical power of 6.7 mW, corresponding to an optical fluence of ~ 106.6 **μJ cm^{-2}.** Measured X-ray counts versus distance from the center of the optical beam for (A) I (30-keV PE energy), (B) Si (30-keV PE energy), and (C) Sn (30-keV PE energy), (D) Pb (30-keV PE energy), (E) Pb (5-keV PE energy), (F) O (5-keV PE energy), (G) N (5-keV PE energy), and (H) C (5-keV PE energy). Legends indicate optical exposure time. The qualitative features of the ion distribution after light exposure in $MAPbI_3$ are similar to those observed in $FAPbI_3$ as discussed in the main text.

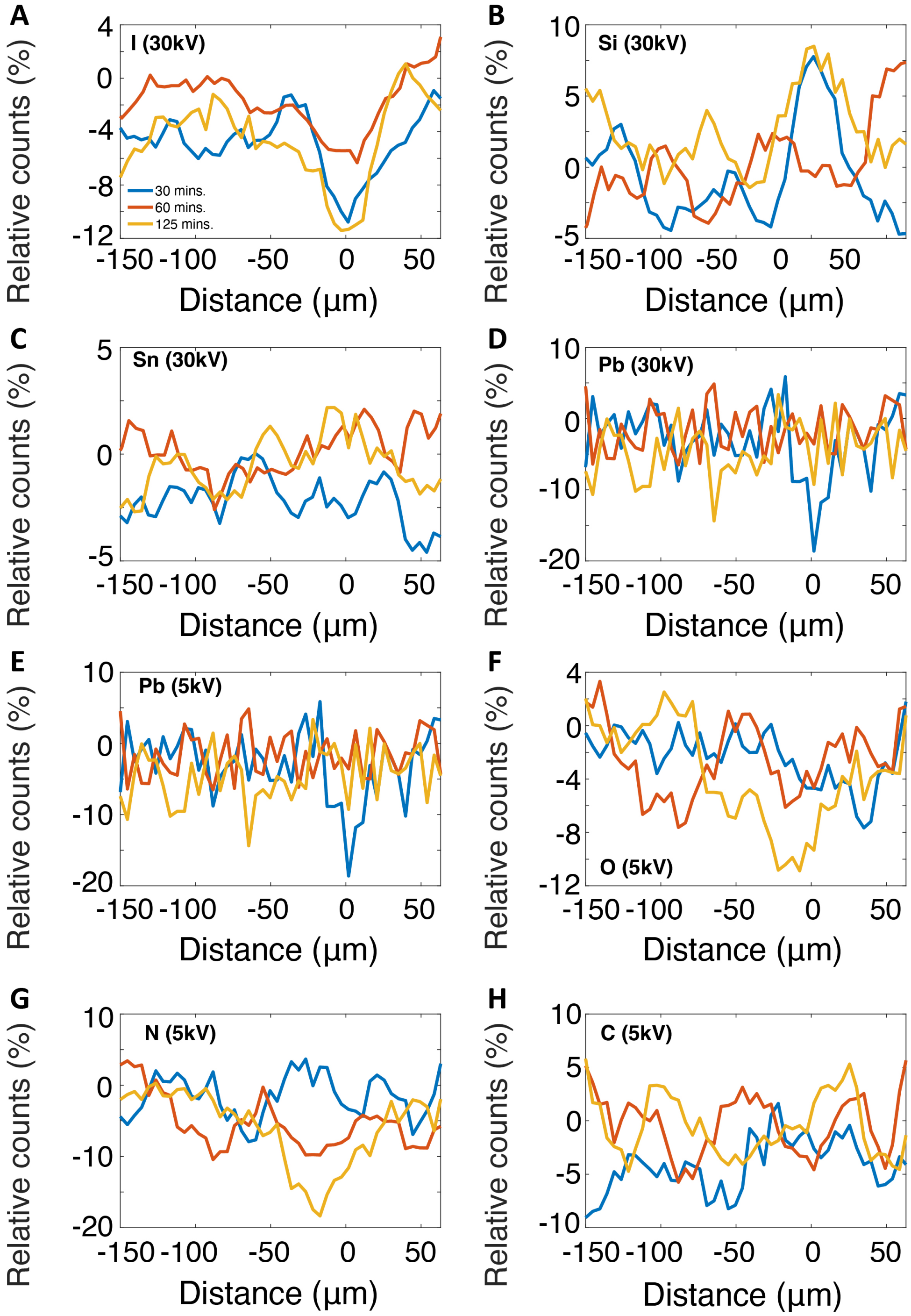

Figure S13. Additional EDS linescan results in $MAPbI_3$ at optical power of 4.1 mW, corresponding to an optical fluence of $\sim 66.0\ \mu$J cm^{-2}. Measured X-ray counts versus distance from the center of the optical beam for (A) I (30-keV PE energy), (B) Si (30-keV PE energy), and (C) Sn (30-keV PE energy), (D) Pb (30-keV PE energy), (E) Pb (5-keV PE energy), (F) O (5-keV PE energy), (G) N (5-keV PE energy), and (H) C (5-keV PE energy). Legends indicate optical exposure time.

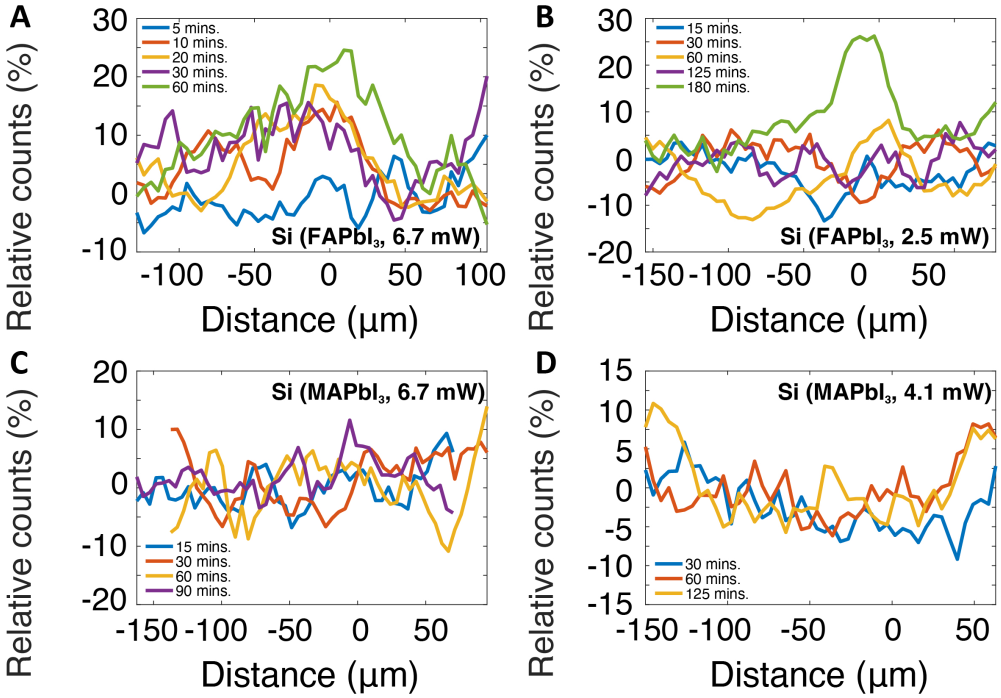

Figure S14. EDS linescan results for silicon at 5-kV in $FAPbI_3$ and $MAPbI_3$

Measured EDS linescan for silicon at optical power of (A) 6.7 mW (optical fluence: $\sim$ 106.6 μJ cm^{-2}), (B) 2.5 mW (optical fluence: $\sim$ 39.8 μJ cm^{-2}) in $FAPbI_3$. Also shown are those at optical power of (C) 6.7 mW (optical fluence: $\sim$ 106.6 μJ cm^{-2}), and (D) 4.1 mW ($\sim$ 66.0 μJ cm^{-2}) in $MAPbI_3$. Legends indicate the optical exposure time.

G. EDS mapping of $FAPbI_3$-on-gold sample after light exposure

As discussed in the main text, we observed increased silicon and tin X-ray counts in the EDS mapping after light exposure and we attributed this observation to potential migration of silicon and tin ions from the substrate. An alternative possibility is that light exposure caused a change in the density of the perovskite film or created cracks and gaps in the perovskite film that allowed more probing electrons to reach the substrate. To investigate this possibility, we conducted a comparative study of a $FAPbI_3$ sample deposited on a 80-nm gold-coated FTO glass substrate. If indeed the density of the perovskite is changed after exposure or light-induced structural changes create gaps in the film so that the electron beam can excite the substrate more, we would expect an increased X-ray counts associated with the gold characteristic line. The results are shown here in Fig. S15, where the changes in the X-ray counts normalized to the background values associated with silicon (K_α band, 1.74 keV), tin (L_α band, 3.44 keV for 30 keV PE and M band, 0.69 keV for 5 keV PE) and gold (M band, 2.12 keV) after light exposure (fluence: 105 μJ cm^{-2}, 10 minutes exposure) are displayed. The shaded area denotes the exposed region in the sample. First of all, the X-ray counts associated with gold were minimally changed within the background fluctuation after the light exposure. The X-ray counts associated with tin showed a similar behavior. These observations suggest that it is unlikely that the perovskite film passes more electrons to the substrate after light exposure. Surprisingly, we noticed the X-ray counts associated with silicon when probed by 30 keV PEs were still significantly enhanced in this case. While we do not expect silicon ions to migrate across the FTO and gold layers, the observed enhancement likely suggests an enrichment of silicon in FTO and even gold within the exposed region. Unfortunately, at this stage we do not have access to characterization techniques that can directly prove the presence of silicon and/or tin ions inside perovskite after light exposure.

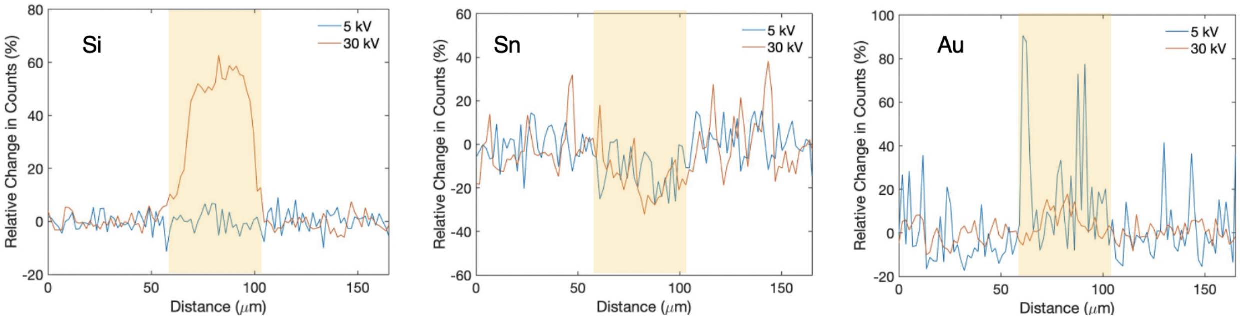

Figure S15. EDS mapping of a $FAPbI_3$-on-gold sample after light exposure. The sample was exposed to light with a fluence of 105 μJ cm^{-2} for 10 minutes. The left, center and right panels show the changes in the X-ray counts normalized to the background values associated with silicon (K_α band, 1.74 keV), tin (L_α band, 3.44 keV for 30 keV PE and M band, 0.69 keV for 5 keV PE) and gold (M band, 2.12 keV) after light exposure. The shaded area denotes the exposed region in the sample.

H. Additional Cathodoluminescence Data

1. *Cathodoluminescence Microscopy of Pristine $MAPbI_3$ after in situ 1.3 mW pulsed laser exposure*

At higher laser powers (1.3 mW compared to 100μW in the main text), substantial differences are seen in the $MAPbI_3$ CL spectrum images, as seen in Fig. S16. Even at substantially shorter exposure times, the excitonic luminescence is completely suppressed in the vicinity of the laser spot as a result of thermal degradation processes. The PbI_2 CL is also completely suppressed in the vicinity of the laser spot, just as it was for lower laser powers.

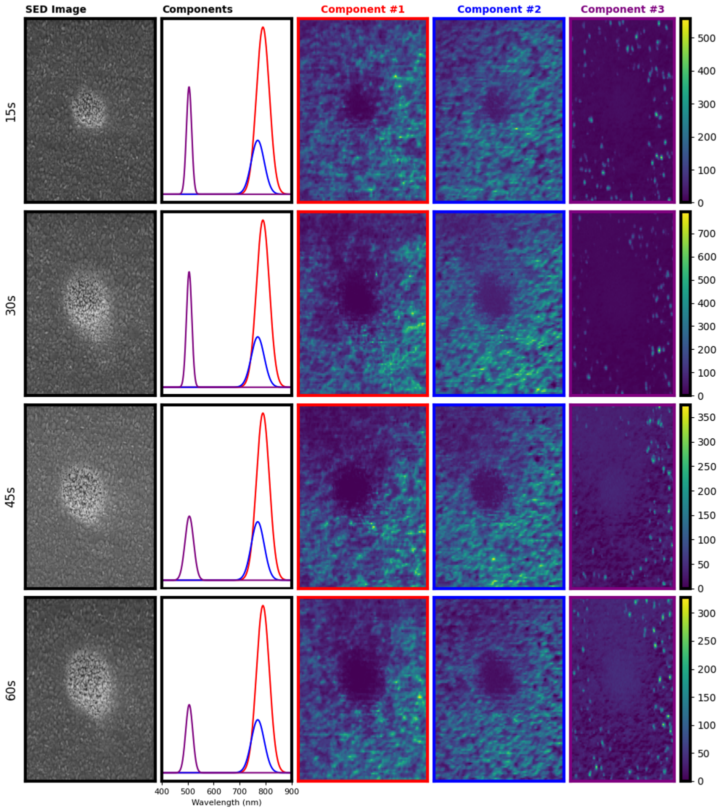

Figure S16. Cathodoluminescence analysis of $MAPbI_3$ film after *in situ* 1.3 mW pulsed laser exposure. Correlated SE images (first column) and CL NMF decomposition (columns 2 to 5) after exposure to a 495 nm, 1.3 mW fs laser source focused to a 5 μm spot size for laser exposure times of 15-60s. Three spectral components are used in the NMF decomposition to highlight the $MAPbI_3$ exciton CL (component 1; red), the partially degraded $MAPbI_3$ exciton CL (component 2; blue), and the PbI_2 CL (component 3; purple). The horizontal field width of all images is 9.1 μm.

2. Cathodoluminescence Microscopy of Pristine $FAPbI_3$ after in situ 1.3 mW pulsed laser exposure

The pristine $FAPbI_3$ films exhibited no PbI_2 CL but substantially greater spatial heterogeneity in the excitonic CL spectrum images compared with the pristine $MAPbI_3$ described in the main text. Figure S17 highlights the response of a $FAPbI_3$ film to increasing laser exposure times for a 495 nm, 1.3 mW fs laser source. While the increased SE brightness and changes in morphology are consistent with that observed for $MAPbI_3$, it is hard to conclusively determine the effect of laser exposure on the CL response because of the intrinsic spatial heterogeneity present in the pristine film.

3. Cathodoluminescence Microscopy of hybrid 97% $FAPbI_3$/3% $MAPbBr_3$ perovskite film after in situ 300 μW pulsed laser exposure

Like the pristine $MAPbI_3$ films, hybrid 97% $FAPbI_3$/3% $MAPbBr_3$ perovskite films exhibited isolated, low intensity PbI_2 CL with reasonably homogeneous excitonic CL. The hybrid films were more robust to laser exposure than the other films, and they exhibited no measurable change in morphology or optical properties for laser powers below 300 μW. After exposure to a 495 nm, 300 μW fs laser source focused to a 5 μm spot size for exposure times of 120-3600s, they exhibited a slight increase in SE brightness and surface microstructuring, and the excitonic and PbI_2 CL were moderately suppressed in the area surrounding the laser spot, as shown in Fig. S18.

4. Cathodoluminescence Microscopy of hybrid 97% $FAPbI_3$/3% $MAPbBr_3$ perovskite film after in situ 1.25 mW pulsed laser exposure

At higher laser powers, thermal processes again emerged, resulting in some intriguing effects in the hybrid 97% $FAPbI_3$/3% $MAPbBr_3$ perovskite film. As shown in Fig. S19, we did not observe any statistically significant increase in the intensity of the intermediate phase CL as a function of laser exposure or position. However, while the band-edge luminescence is completely suppressed in the microstructured area, the band-edge CL intensity increases by up to a factor of 20 in the bright ring surrounding the laser spot (with a monotonic increase in CL intensity as a function of laser exposure time). Note that this film was prepared separately from the film characterized in Fig. S18, and it exhibited substantially reduced PbI_2 CL compared with that film.

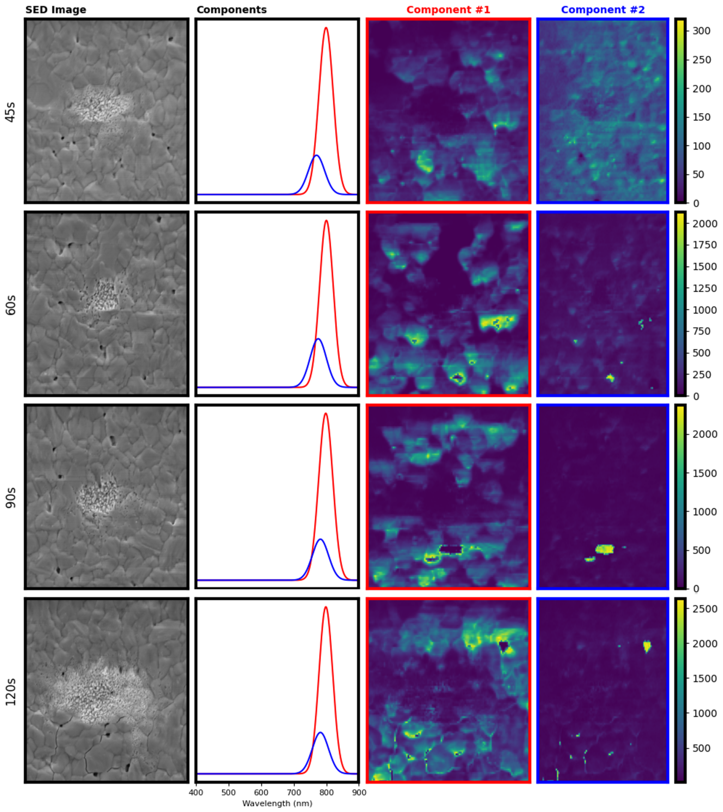

Figure S17. Cathodoluminescence analysis of $FAPbI_3$ film after *in situ* 1.3 mW pulsed laser exposure. Correlated SE images (first column) and CL NMF decomposition (columns 2 to 5) after exposure to a 495 nm, 1.3 mW fs laser source focused to a 5 μm spot size for laser exposure times of 15-60s. No PbI_2 CL is observed anywhere on the film, and two NMF components are sufficient to achieve a 99% explained variance ratio. The horizontal field width of all images is 9.1 μm.

5. *Cathodoluminescence Data after CW Laser Exposure*

Additional NMF decompositions of CL spectrum images acquired for a hybrid 97% $FAPbI_3$/3% $MAPbBr_3$ perovskite film exposed to an *in situ* 532 nm, CW, 3 mW laser focused to a 5 μm spot are shown in Fig. S20. The results are highly consistent with the CL spectrum images acquired after pulsed laser excitation and illustrated in Fig. S19. In particular, the perovskite band-edge luminescence is completely suppressed under laser excitation, but a bright ring with up to an order of magnitude enhancement of band-edge luminescence is observed surrounding the exposed area.

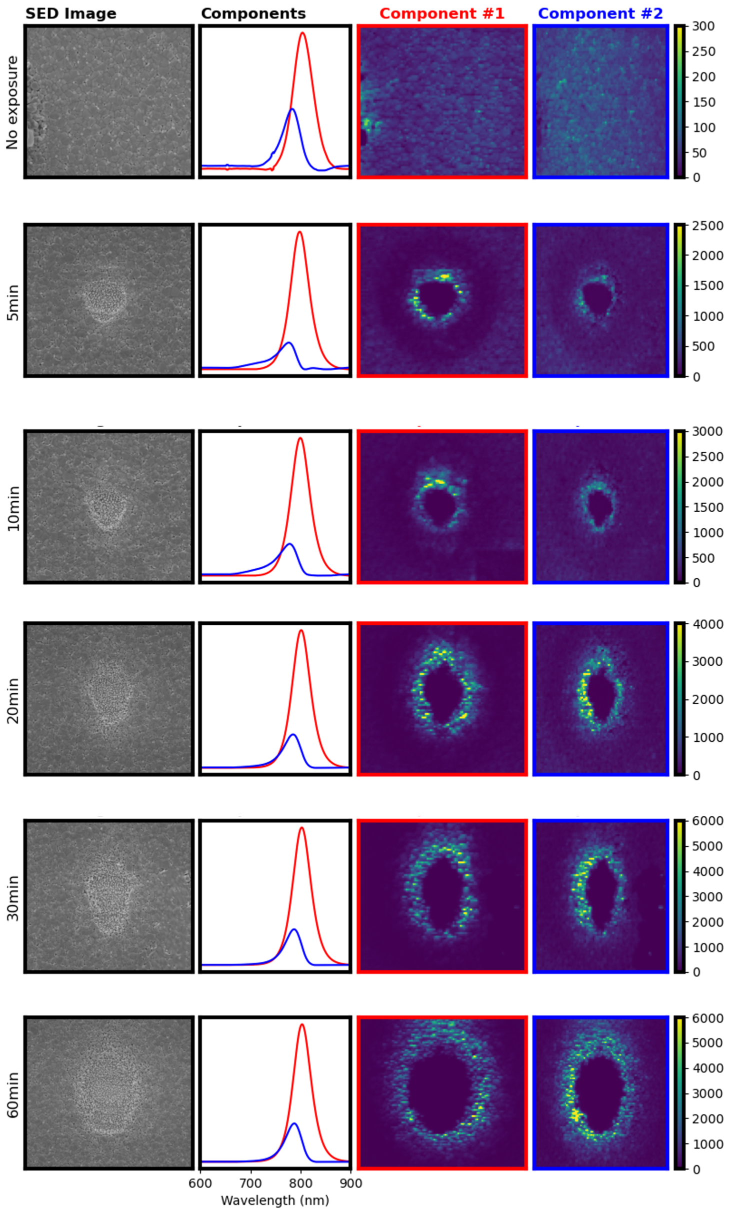

Figure S18. Cathodoluminescence analysis of hybrid 97% $FAPbI_3$/3% $MAPbBr_3$ perovskite film after *in situ* 300 μW pulsed laser exposure Correlated SE images (first column) and CL NMF decomposition (columns 2 to 5) after exposure to a 495 nm, 300 μW fs laser source focused to a 5 μm spot size for laser exposure times of 120-3600s. The horizontal field width of all images is 22.3 μm.

Also, no PbI_2 band-edge luminescence is observed before or after laser exposure, and the laser exposure doesn't change the relative abundance of the intermediate degraded perovskite phase.

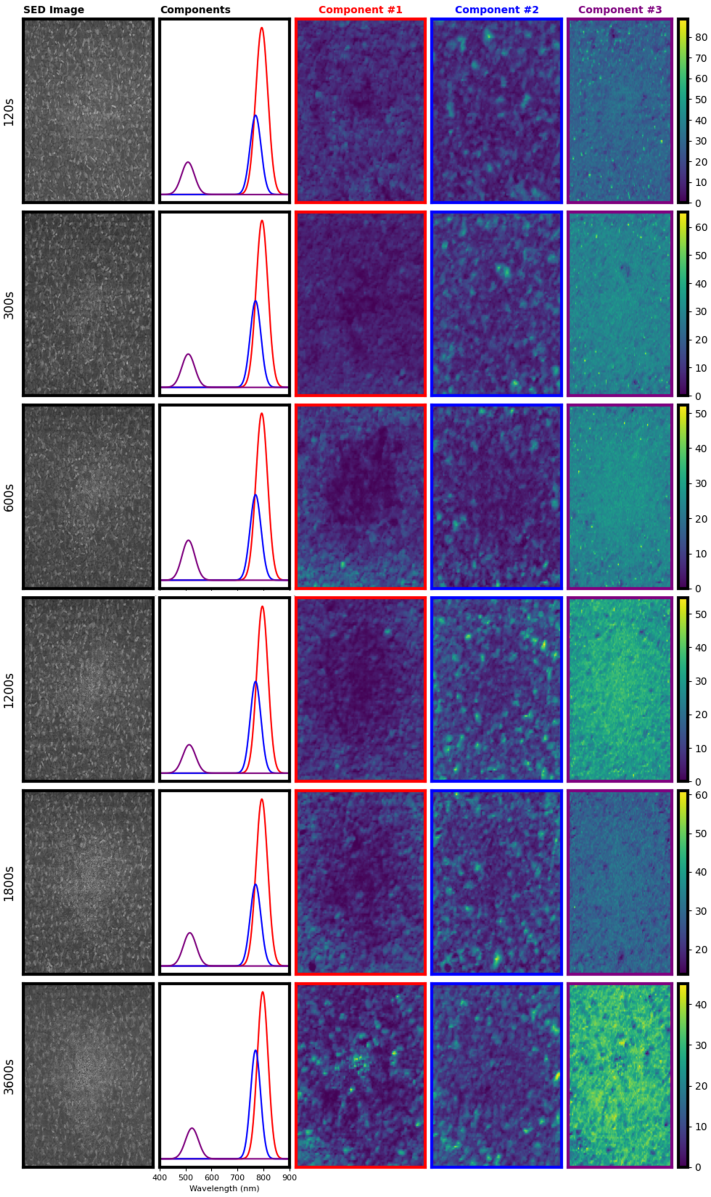

Figure S19. Cathodoluminescence analysis of hybrid 97% $FAPbI_3$/3% $MAPbBr_3$ perovskite film after *in situ* 300 μW pulsed laser exposure Correlated SE images (first column) and CL NMF decomposition (columns 2 to 4) after exposure to a 495 nm, 300 μW fs laser source focused to a 5 μm spot size for laser exposure times of 300-3600s. The horizontal field width of all images is 21.1 μm.

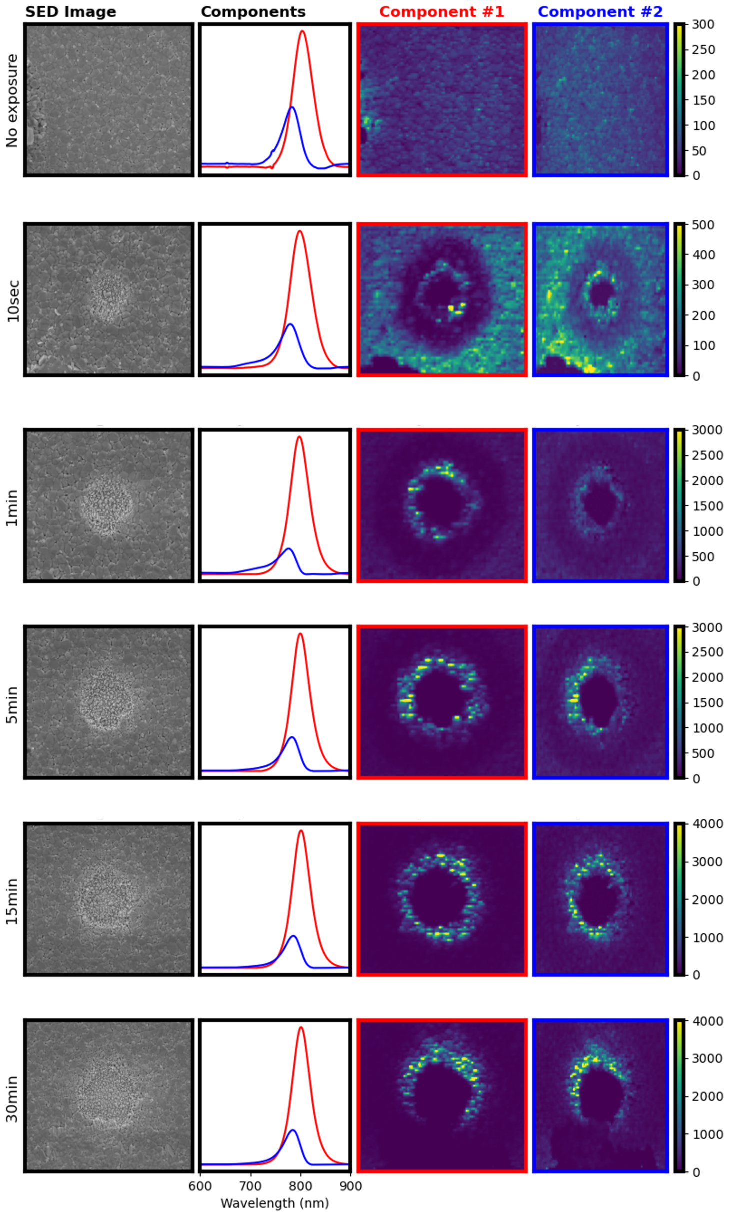

Figure S20. Cathodoluminescence analysis of 97% $FAPbI_3$/3% $MAPbBr_3$ after *in situ* exposure with a 532 nm, CW, 3 mW CW laser source. Concurrent SE images (first column) and CL NMF decompositions for a pristine sample and for samples exposed to the laser for 10 s, 1 minute, 5 minutes, 15 minutes, and 30 minutes. The two spectral components of each NMF decomposition are illustrated in the second column, and the intensity maps for each of those components are illustrated in the third and fourth columns. The color bars highlight the increase in band-edge CL intensity as a function of laser exposure time. All images have a 22.2 μm horizontal field width.

I. Cathodoluminescence Data after CW Laser Exposure of environmentally degraded $FAPbI_3$

After extended exposure to ambient conditions for a period of several weeks, $FAPbI_3$ and $MAPbBr_3$ films exhibit substantial degradation, with no visible band-edge luminescence remaining. The first row of Fig. S21 illustrates a prototypical CL image of an environmentally degraded perovskite film (in this case, a $FAPbI_3$ film). Bright PbI_2 luminescence is visible in localized regions (component 1), but no band edge perovskite luminescence is visible. Intriguingly, as seen in the second row of Fig. S21, after 10s exposure to a CW, 532 nm, 3 mW laser source, the PbI_2 luminescence near the exposed area is completely suppressed, and a bright ring of $FAPbI_3$ luminescence around the exposed area emerges. This result points to the potential impact of laser induced ion migration in perovskite thin films as a resource for remedying environmental degradation. An alternative explanation is the structural phase transition well known in $FAPbI_3$: the black α-phase can degrade into the yellow δ-phase after environmental exposure, and the inverse transform can happen at an elevated temperature [2]. However, the yellow δ-phase is known to exhibit a broadband luminescence from 400 nm to 650 nm [3], which was not observed in our experiment, indicating that this structural phase transition was not the reason for our observed recovery of the band-edge $FAPbI_3$ emission.

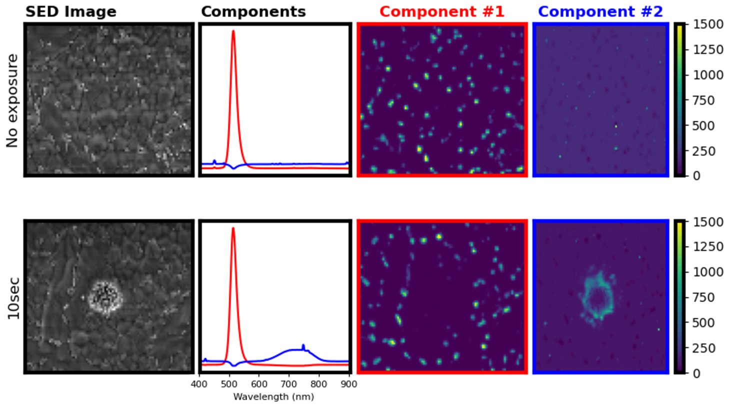

Figure S21. Cathodoluminescence analysis of $FAPbI_3$ film after complete environmental degradation. After extensive environmental degradation, the $FAPbI_3$ film exhibits only heterogeneous PbI_2 luminescence with no band edge luminescence visible in the NMF decomposition of a CL spectrum image acquired prior to laser irradiation (first row). After 10 s *in situ* exposure with a 532 nm, CW, 3 mW CW laser source, the PbI_2 luminescence near the laser spot is completely suppressed, and the perovskite luminescence is partially recovered, as seen in component two of the NMF reconstruction (second row). Both datasets have a 14.6 μm horizontal field width.

SUPPLEMENTARY NOTE 2: MONTE CARLO SIMULATION OF PRIMARY ELECTRON PENETRATION THROUGH THE SPECIMEN

We simulated the trajectory of primary electrons across the sample considered in this study using the computer program CASINO (Monte Carlo Simulation of Electron Trajectory in Solids) [4]. As given in the inset of Fig. S22B, we considered our sample geometry containing three layers (two thin layers and one thick layer). The corresponding thin layers are 500-nm $MAPbI_3$ or 800-nm $FAPbI_3$, and 130-nm SnO_2. For modeling purposes, we treated the SiO_2 substrate as a semi-infinite, despite its actual thickness of ~ 3 mm. Then, we assumed that the electron beam (total number of electrons: 10000, electron beam radius: 10 nm) was incident on the specimen with initial energy of 30 keV and 5 keV, depending on the primary electron acceleration voltages in our SEM settings, and that they underwent successive collision events inside the specimen. Figure S22A shows our simulation results for $MAPbI_3$ layer in which many of the primary electrons penetrate through the entire $MAPbI_3$ layer and into the substrate at 30 keV. In contrast, the primary electrons with initial kinetic energies of 5 keV were mostly confined within $\lesssim$ 200 nm near the surface. The simulation results of $FAPbI_3$ is shown in Fig. 1C and D in the main text.

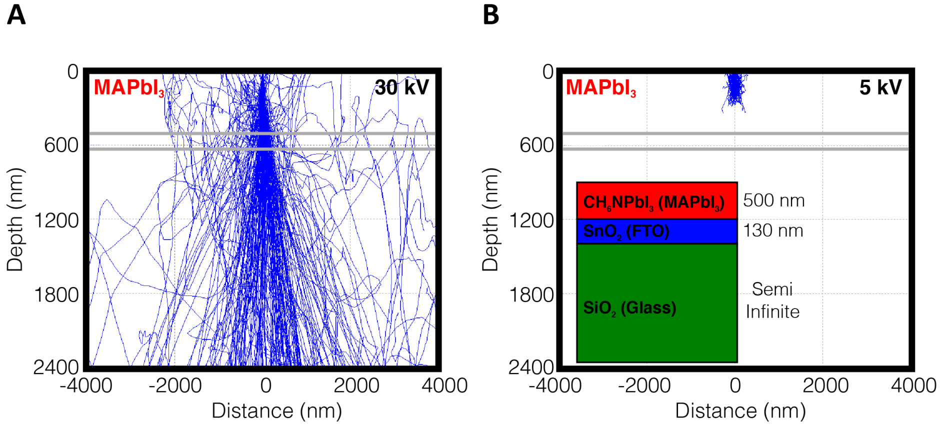

Figure S22. Monte Carlo Simulation of Electron Trajectory in $MAPbI_3$. The simulated trajectories of primary electrons with energies of (A) 30 keV, and (B) 5 keV in a $MAPbI_3$ sample. While the primary electrons can penetrate the entire sample stack at 30 keV, those at 5 keV are mostly confined within 200 nm near the $MAPbI_3$ surface. The interfaces between $MAPbI_3$ and FTO, and between FTO and the glass substrate are labeled with gray lines. The inset in (B) shows the structure of the $MAPbI_3$ sample stack.

SUPPLEMENTARY NOTE 3: ESTIMATION OF X-RAY SELF-ABSORPTION DEPTH

This section presents our estimation of the X-ray self-absorption depth. First, we consider the elemental specific energy bands of the X-rays emitted from the specimen, which is presented in Tab. SI. Then, given the particular energy band, the total mass absorption coefficient by a certain element (μ/ρ) was obtained using an established database [5], where μ is the linear absorption coefficient with a dimension of 1/length (the inverse of the absorption depth) and ρ is the elemental density. For a pure elemental material, the total atomic absorption cross section σ_a can be calculated from the mass absorption coefficient by:

$$\sigma_a = \frac{A}{N_A}\frac{\mu}{\rho}, \tag{S1}$$

where A is the atomic weight and N_A is the Avogadro's constant. For a compound material, the mass absorption coefficient can be obtained from the sum of the absorption cross sections of the constituent atoms by [6]:

$$\left(\frac{\mu}{\rho}\right)_{\text{compound}} = \frac{N_A}{A_{\text{tot}}}\sum_i x_i\sigma_{ai}, \tag{S2}$$

where the molecular weight of a compound containing x_i atoms of type i is $A_{\text{tot}} = \sum_i x_i A_i$. This equation does not include interactions among the constituent atoms, but is generally applicable for X-ray photon energies above 30 eV and sufficiently far from absorption edges [6]. Given their large atomic masses, lead and iodine are the major contributors to the total absorption cross section in both $MAPbI_3$ and $FAPbI_3$. Combining Eqns. S1 and S2 and data from the database, the absorption lengths of the characteristic X-ray energies in $MAPbI_3$ were calculated and listed in Table SI. The values in $FAPbI_3$ should be similar given the same lead and iodine composition. As shown in Table SI, for lead, iodine and tin, the X-ray absorption depth far exceeds the sample thickness ($\sim$ 500 nm for $FAPbI_3$; $\sim$ 800 nm for $MAPbI_3$). Therefore, the probing depth of the EDS measurement of these elements was only determined by the penetration depth of the primary electrons. On the other hand, the X-ray absorption depths of carbon, nitrogen and oxygen were smaller than the sample thickness, and thus the EDS probe was only sensitive to their distributions near the sample surface. The situation of silicon is in the middle: even with 30-keV primary electrons, the EDS was only sensitive to silicon distribution within roughly 400 nm near the surface due to the fact that X-rays emitted deeper inside the sample cannot escape and be collected by the EDS detector.

Table SI. Element specific X-ray emission bands detected in our experiments and their absorption length in $MAPbI_3$

Element	Photon energy (keV)	Characteristic principal line	Absorption length in $MAPbI_3$ (nm)
Pb	2.35	$M_{\alpha1}$	854
I	3.94	$L_{\alpha1}$	2780
C	0.277	K_{α}	148
N	0.392	K_{α}	173
O	0.525	K_{α}	264
Si	1.74	K_{α}	424
Sn	3.44	$L_{\alpha1}$	1970

SUPPLEMENTARY NOTE 4: FITTING PROCEDURES FOR DETERMINING DIFFUSIVITY

This section presents our procedures for determining the diffusivity of iodine ions. First, the profiles of the horizontal linescans were normalized, as shown in Fig. S23A. Next, due to emerging asymmetry of the profiles and background noise, the normalized profiles were divided into right (Fig. S23B-F) and left (Fig. S24A-F) sections, which were separately analyzed using a one-dimensional Gaussian model. Although we carefully attempted to fit the left profiles in Fig. S24A-F, we found that the data and fit exhibit slight discrepancies particularly near the origin, leading to more scattered resulting fitted radii, thus complicating our analysis, as shown in Fig. S24F. In contrast, we found an improved agreement between the right profiles and the Gaussian fits, as shown in Fig. S23B-F. As a consequence, the trend for the radii versus exposure time was less scattered compared to those for the left profiles (see Fig. S24F). Then, once the fitted radii were determined at each exposure time, we additionally fitted the diffusivity assuming one-dimensional (horizontal) diffusion [7]. The results of fitting using the one-dimensional diffusion model are presented as dashed lines in Fig. S24F. We here note that despite more scattered Gaussian-fitted radii emerging for the left profiles, the fitted values of the diffusion coefficients from left and right profiles were reasonably close to each other. We also note that the calculation here is meant to be an order-of-magnitude estimation, since the real ion migration process is more complicated than the simple one-dimensional diffusion model used here.

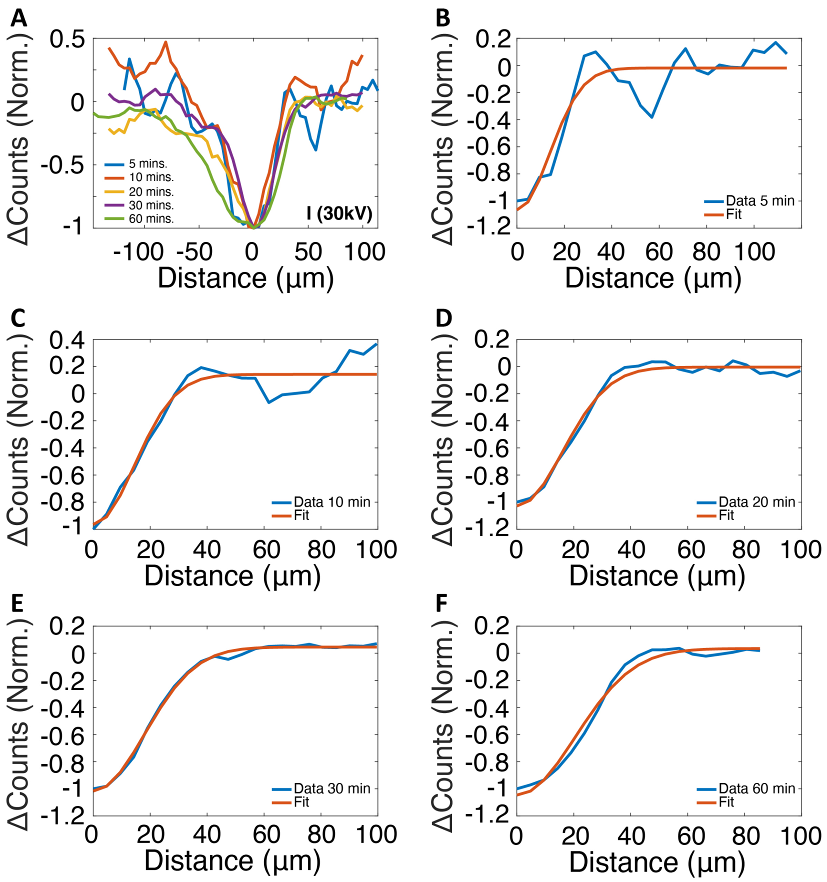

Figure S23. Analysis of horizontal EDS linescan profile for iodine using the right-half profiles (optical fluence: $\sim 106.6\ \mu$J cm^{-2}). (A) The normalized difference counts versus distance at various optical exposure times ranging from 5 minutes to 60 minutes. The profile was divided into two at the horizontal origin, in which right fractional profiles under optical exposure for (B) 5 minutes, (C) 10 minutes, (D) 20 minutes, (E) 30 minutes, and (F) 60 minutes were analyzed (blue solid lines). The corresponding profile was fitted using one-dimensional Gaussian models (red solid lines), in which we found a reasonable agreement for all the data considered here.

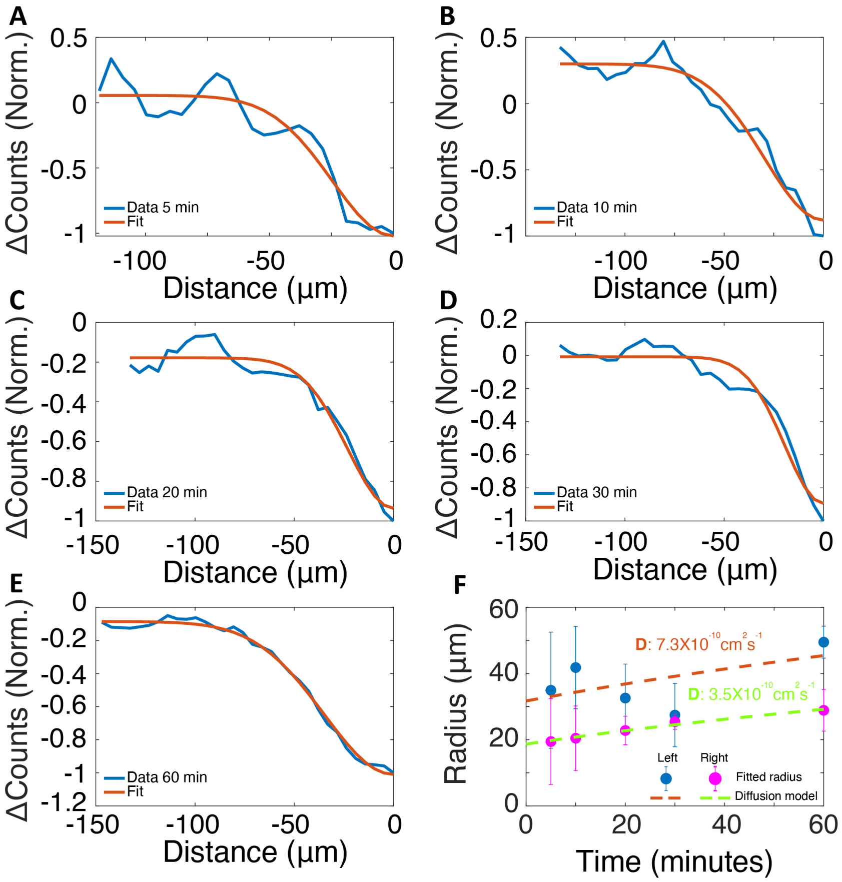

Figure S24. Analysis of horizontal EDS linescan profile for iodine using left-half profiles (optical fluence: ~ 106.6 μJ cm^{-2}). The analyzed left fractional profiles under optical exposure for (A) 5 minutes, (B) 10 minutes, (C) 20 minutes, (D) 30 minutes, and (E) 60 minutes were analyzed (blue solid lines), along with one-dimensional Gaussian model fits (red solid lines). The discrepancies between the data and Gaussian fit can be seen due to asymmetry of the profiles and background noise. (F) The Gaussian-fitted radii versus the optical exposure time determined using left and right profiles are shown. The error bars indicate numerically determined 95% confidence intervals. The dashed lines indicate best fits determined using the one-dimensional diffusion model, from which the diffusivities are annotated near each fit.

SUPPLEMENTARY NOTE 5: ESTIMATION OF LASER-INDUCED TEMPERATURE RISE

Since we used a pulsed laser source in our experiment, we need to estimate both the transient temperature rise after each laser pulse is absorbed and the steady-state temperature rise induced by the periodic pulse train. The transient temperature rise can be estimated in the following way. The highest optical fluence used in our experiment was $\sim 100\mu\text{J cm}^{-2}$. The reflectance of $MAPbI_3$ and $FAPbI_3$ is about 25% at 515 nm [8, 9]. Given the optical absorption depth of roughly 100 nm in both $MAPbI_3$ and $FAPbI_3$ [8–10] at 515 nm, the volumetric heat capacity of $MAPbI_3$ ($\sim 1.28 \times 10^6$ J $\text{m}^{-3}\text{K}^{-1}$) [11], the transient temperature rise after each pulse is absorbed can be estimated to be:

$$\Delta T_{\text{transient}} = \frac{0.75 \times 100\,\mu\text{J cm}^{-2}}{100\,\text{nm} \times 1.28 \times 10^6\,\text{J m}^{-3}\text{K}^{-1}} = 5.8\,\text{K}. \tag{S3}$$

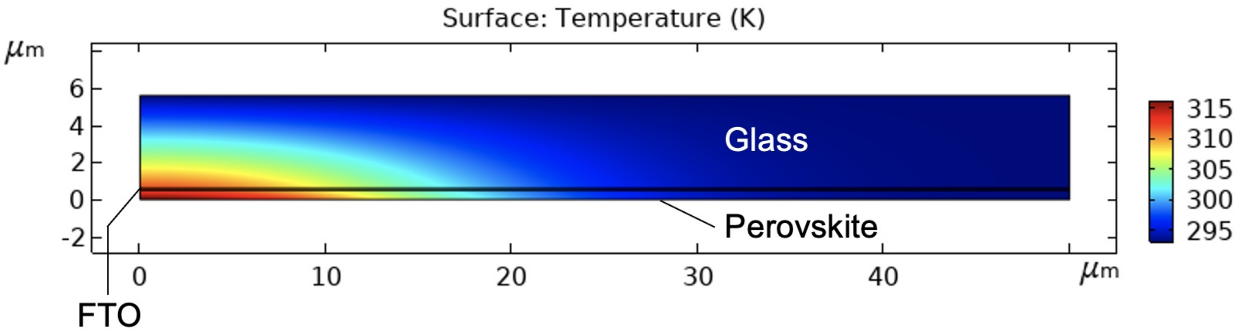

Figure S25. Finite element simulation of the laser-induced steady-state temperature rise. The simulated sample stack consisted of 500-nm $MAPbI_3$, 100-nm FTO and 5-μm glass. The environmental temperature was set at 293 K. The temperature distribution is coded in the color bar.

We estimated the steady-state temperature rise using a finite element simulation (COMSOL Multiphysics). We used the thermal conductivity values for FTO: 4 W $\text{m}^{-1}\text{K}^{-1}$ [12], and glass substrate: 1.2 W $\text{m}^{-1}\text{K}^{-1}$. The steady-state temperature rise was obtained by simulating the heating effect of a continuous-wave source with the same average power (6.7 mW was the highest optical power used in our study). An optical beam $1/e^2$ radius of 25 μm and an optical absorption depth of 100 nm were used in the simulation. The simulated sample stack consisted of 500-nm $MAPbI_3$, 100-nm FTO and semi-infinite glass (5-μm thick in the simulation). The environmental temperature was set at 293 K. The simulated temperature distribution is shown in Fig. S25. From

the simulation, the highest temperature at the center of the laser beam reached 316 K, which was 23 K higher than the initial temperature. Therefore, the overall laser-induced temperature rise (including both transient and steady state rises) is $\Delta T < 30$ K. This estimation suggests that the impact of laser heating to our observed ion migration is much smaller compared to a recent observation on the heat-induced migration effect, in which the temperature rise was on the order of ~ 100 degrees [13].

For the CL measurement, due to the smaller beam size (5 μm diameter), the simulation suggested an optical power below 300 μW is required to limit the temperature rise below 30 K.

Supplementary References

[1] W.-C. Lin, W.-C. Lo, J.-X. Li, Y.-K. Wang, J.-F. Tang, and Z.-Y. Fong, In situ XPS investigation of the X-ray-triggered decomposition of perovskites in ultrahigh vacuum condition, npj Materials Degradation **5**, 13 (2021).

[2] C. C. Stoumpos, C. D. Malliakas, and M. G. Kanatzidis, Semiconducting tin and lead iodide perovskites with organic cations: phase transitions, high mobilities, and near-infrared photoluminescent properties, Inorganic Chemistry **52**, 9019 (2013).

[3] F. Ma, J. Li, W. Li, N. Lin, L. Wang, and J. Qiao, Stable α/δ phase junction of formamidinium lead iodide perovskites for enhanced near-infrared emission, Chemical Science **8**, 800 (2017).

[4] P. Hovington, D. Drouin, and R. Gauvin, CASINO: A new Monte Carlo code in C language for electron beam interaction — Part I: Description of the program, Scanning **19**, 1 (1997), https://onlinelibrary.wiley.com/doi/pdf/10.1002/sca.4950190101.

[5] B. L. Henke, E. M. Gullikson, and J. C. Davis, X-ray interactions: photoabsorption, scattering, transmission, and reflection at E= 50-30,000 eV, Z= 1-92, Atomic Data and Nuclear Data Tables **54**, 181 (1993).

[6] A. Thompson, D. Attwood, E. Gullikson, M. Howells, K.-J. Kim, J. Kirz, J. Kortright, I. Lindau, P. Pianetta, A. Robinson, *et al.*, X-ray data booklet. Lawrence Berkeley National Laboratory, University of California, Berkeley, CA (2001).

[7] B. Liao, E. Najafi, H. Li, A. J. Minnich, and A. H. Zewail, Photo-excited hot carrier dynamics in hydrogenated amorphous silicon imaged by 4D electron microscopy, Nature Nanotechnology **12**, 871 (2017).

[8] M. Shirayama, H. Kadowaki, T. Miyadera, T. Sugita, M. Tamakoshi, M. Kato, T. Fujiseki, D. Murata, S. Hara, T. N. Murakami, *et al.*, Optical transitions in hybrid perovskite solar cells: ellipsometry, density functional theory, and quantum efficiency analyses for $CH_3NH_3PbI_3$, Physical Review Applied **5**, 014012 (2016).

[9] M. Kato, T. Fujiseki, T. Miyadera, T. Sugita, S. Fujimoto, M. Tamakoshi, M. Chikamatsu, and H. Fujiwara, Universal rules for visible-light absorption in hybrid perovskite materials, Journal of Applied Physics **121**, 115501 (2017).

[10] J. Y. Kim, J.-W. Lee, H. S. Jung, H. Shin, and N.-G. Park, High-efficiency perovskite solar cells, Chemical Reviews **120**, 7867 (2020).

[11] T. Haeger, R. Heiderhoff, and T. Riedl, Thermal properties of metal-halide perovskites, Journal of Materials Chemistry C **8**, 14289 (2020).

[12] D. H. Olson, C. M. Rost, J. T. Gaskins, C. J. Szwejkowski, J. L. Braun, and P. E. Hopkins, Size effects on the cross-plane thermal conductivity of transparent conducting indium tin oxide and fluorine tin

oxide thin films, IEEE Transactions on Components, Packaging and Manufacturing Technology **9**, 51 (2018).

[13] M. Lai, A. Obliger, D. Lu, C. S. Kley, C. G. Bischak, Q. Kong, T. Lei, L. Dou, N. S. Ginsberg, D. T. Limmer, *et al.*, Intrinsic anion diffusivity in lead halide perovskites is facilitated by a soft lattice, Proceedings of the National Academy of Sciences **115**, 11929 (2018).
